# Trends in Ectopic Pregnancies in Eastern Saudi Arabia

**DOI:** 10.1155/2013/975251

**Published:** 2013-02-25

**Authors:** Haifa Abdulaziz Al-Turki

**Affiliations:** ^1^College of Medicine, University of Dammam, Dammam 31442, Saudi Arabia; ^2^King Fahd Hospital of the University, Al-Khobar 31952, Saudi Arabia

## Abstract

*Background*. The objective of this study was to estimate trends in ectopic pregnancies (EP) in a tertiary care center of Eastern Saudi Arabia. *Method*. Information about patients with ectopic pregnancies who had been admitted to King Fahd Hospital of the University, AlKhobar, between January 2000 and 31 December 2011 was collected from a computerized hospital registry. Age-specific ectopic pregnancy incidence was calculated. The data was analyzed using SPSS (Statistical Package for the Social Sciences), version 14.0 (Chicago, IL, USA). *Results*. There were 274 EPs during the study period; the yearly incidence in terms of 24,098 deliveries was 1.19%. The average age was 28.99 Å 5.62 years. During a three-year period (2000–2002), the incidence was 0.92%; from 2003 to 2005, the incidence was 1.01%; from 2006 to 2008, the incidence was 1.51%; and from 2009 to 2011, the incidence was 1.35%. Age-adjusted ectopic pregnancy incidence rates steadily increased from 92.23 per 10,000 women years during the period 2000–2002 to 149.408 during the 2006–2008 period; since then, it has declined to 110.313 per 10,000 women years. *Conclusions*. Our study reveals that the incidence of EP has decreased from what it had been during the mid-2000s but has remained significantly elevated when compared to the early 2000s.

## 1. Introduction

An ectopic pregnancy occurs outside the uterus and is a relatively common condition among women of childbearing age. The reported incidence of EP in the Western world is reported to be in the range of 1–3% of all pregnancies [1, 2]. Studies from Saudi Arabia put the prevalence at between 0.58–1.13% [3–5]. Pregnancy-related maternal mortalities in the first trimester account for 4 to 10 percent of all pregnancy-related deaths [6, 7].

In vitro fertilization (IVF) has long been recognized as a serious risk factor for EP [8, 9]; in the recent past, the number of IVF pregnancies has increased, but the same reports indicate either stable or steadily decreasing incidence [10–12]. Trabert et al. (2011) recently reported an increasing trend of EP during the last 15 years [13]. A similar picture has emerged from Saudi Arabia, as previous studies have reported an incidence of 0.58–0.74%, Three, 4-, 14- and an 11-year analysis reported an incidence of 1.13% [5].

The objective of this study was to determine the incidence of ectopic pregnancy in eastern Saudi Arabia at King Fahd University Hospital, which has remained the referral center for the eastern province of Saudi Arabia for the last 25 years.

## 2. Methodology

The data regarding all patients admitted to King Fahd University Hospital, AlKhobar, between 1 January 2000 and 31 December 2011 with a suspected history of EP and patients with proven EP was collected from a computerized hospital registry by using the International Classification of Diseases (ICD) 9th Revision code 633 during 2000 and 2011. Data on age, parity, present pregnancy history, previous infertility treatment, diagnostic methods and findings, date and type of surgery, and results of histological examinations of removed tissue was retrieved from medical records.

The prevalence of ectopic pregnancy in a 12-year-age period was calculated in reference to the number of registered pregnancies during the study period. The extrauterine ratio was defined as the number of ectopic pregnancies divided by the number of births. The prevalence of first versus repeat ectopic pregnancies and the proportion of patients with first ectopic pregnancies and prior infertility treatments or intrauterine contraceptive device (IUCD) use at the time of the diagnosis were calculated. The data was analyzed using SPSS (Statistical Package for the Social Sciences), version 14.0 (Chicago, Illinois). Data was expressed as a mean ± standard deviation (SD). Statistical significance differences between groups were determined with a Student's *t*-test and *P* values of 0.05; a confidence interval (CI) of 95% was considered significant.

## 3. Results 

**Figure 1:**
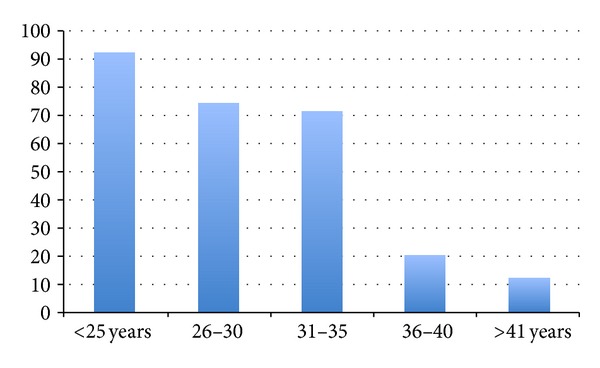
Age distribution of patients.

**Figure 2:**
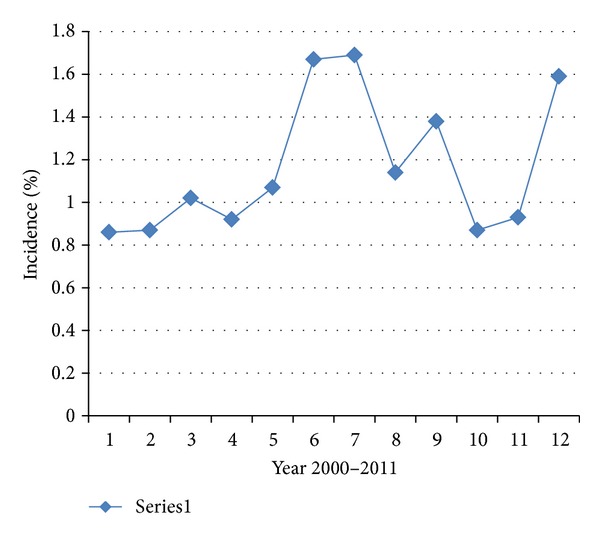
Showing yearly incidence 2000–2011.

There were 274 EPs out of 24,098 deliveries at King Fahd University Hospital, Al-Khobar, for an incidence of 1.14%, with an average patient age of 28.99 ± 5.62 years.  Figure 1 reveals the age distribution of patients, showing that the majority of patients (61.1%) was below the age of 30 years.  Table 1 shows the yearly incidence of EP between 2000 and 2011. Patients were arbitrarily divided into three-year groups. During the three-year period 2000–2002, the incidence of EP was 70/7,589 (0.92%); between 2003–2005, the incidence was 78/7,792 (1.01%); during the period 2006–2008, the incidence was the highest at 73/4,819 (1.51%); and during 2009–2011, the incidence was 53/3,898 (1.35%). Age-adjusted ectopic pregnancy incidence rates steadily increased from 92.238 per 10,000 women years over the period 2000–2002 to 149.408 during the period 2006–2008 and since then declined to 110.313 per 10,000 women years during the 2009–2011 period (Figure 2). The EP rate remained high among women with no previous pregnancies compared with women with previous births.

From the total of women whose data was analyzed, 108 (41.7%) had previous pregnancies compared to 151 (58.3%); that number had a *P* value of 0.0002, 95% CI of <−0.0811. Women who did not become pregnant post-EP were significantly older and were in the majority (28.9 ± 6.9, and 53 women were of 39.59 ± 5.9 years (*P* < 0.001 95% CI of difference <−11.8735)). To arrive at the diagnosis of EP, ultrasonography and B-HCG were conducted for all patients; however, in 170 (65.6%) of the cases, there were sufficient findings suggestive of EP to warrant surgical intervention, whereas in the rest, ultrasound and beta HCG were used to verify the diagnosis. The mean beta HCG level was 6891.65 mIU per mL. Two hundred fifty patients were managed surgically, and 15 patients were treated by methotrexate.

## 4. Discussion

Our study shows that there was a declining trend in terms of ectopic pregnancies after they reached a peak in the mid-2000s; it appears that the incidence is currently on the rise, reaching 1.59% of all pregnancies, even though the number of deliveries per year is less than during that period of time. The number of deliveries is less due to the restriction of non-eligible patients. Studies from the developed countries report a downward trend in the incidence of EP [2, 15–17]. Bakken and Skjeldestad (2006) [18] reported that from 1990 to 1994, a strong decrease in age-adjusted EP incidence was observed in all parity groups. They attributed this decrease to the use of hormonal IUDs rather than the nonhormonal; the presence of genital infections as a high-risk factor for EPs could not be ascertained. An earlier study from our institution concluded that the use of IUDs was a risk factor in reference to EP [5]. In recent years, the younger population of Saudi Arabia has used contraceptive devices at higher rates than women in previous years; secondly, before the facility of IVF was not as popular as of now. In this study, 53 (18.6%) patients were undergoing ART, and 16 (5.83%) used IUDs. Hence, these two factors increased the incidence curve.

Kamwendo et al. (2000) [16] found the increase and decrease incidence of EP was directly related to pelvic inflammatory disease. This was further supported by a study conducted by Bouyer and colleagues in 2003 [19], who concluded that smoking was an added risk. Saudi women generally do not smoke cigarettes, so that factor is unlikely to increase the risk of EP, but there could be a changing pattern in terms of the PID and genital infections; this was not examined in this study. Unfortunately, the data on sexually transmitted infections (STI) in Saudi Arabia is minimal. A recent report revealed that among Saudi women, Chlamydia trachomatis antigen was detected in 10.5% of pregnant and 34.4% of control women, while Neisseria gonorrhoeae among pregnant women was 0.0% and in control women was to 7.8% [20]. The prevalence rates of these infections are higher than the reported rate of 2.9% in Northern Ireland [21], the rate of 4.2% in Japan, and the rate of 4.8% in New Zealand [22, 23]. There is a clear possibility that the cause of the increasing trend of EP could be due to STI, a theory which needs to be corroborated in future studies.

There are conflicting reports regarding the role which induced abortions play in the risk of EP. Holt et al. (1989) [24] reported that induced abortion does not increase the risk of EP. However, Tharaux-Deneux and her colleagues (1998) [25] wrote that induced abortions did significantly increased the risk of EP in succeeding years. In Saudi Arabia, the practice of induced abortions does not exist. Evacuation of the products of conception after spontaneous abortions could injure the endometrium, causing secondary infections and allowing embryos to be implanted elsewhere. In this study, we found that spontaneous abortions were a lower order risk factor; hence, we tend to support the findings of Holt et al. 

Our study has a few limitations, an important one being the restrictions caused by the retrospective nature of our study. It was difficult to estimate all of the influencing causes of EP and their relationship to the increasing trends in terms of that condition. One might speculate that the increasing trend could be related to more infertile couples seeking infertility treatments. One center study for a decade does give the figures which could be relied upon.

In conclusion, the current study finds that there is an increasing trend in terms of EP in the eastern Saudi Arabia. We believe that there is a window of opportunity to ascertain the exact causes and suggest appropriate interventions to reduce this upward trend of EPs.

## Figures and Tables

**Table 1:** Ectopic pregnancy between 2000 and 2011 and yearly deliveries.

Year	Total number of pregnancies	Ectopic pregnancy	Incidence %
2000	2533	22	0.86
2001	2510	22	0.87
2002	2546	26	1.02
2003	2905	27	0.92
2004	2792	30	1.07
2005	2095	35	1.67
2006	1832	31	1.69
2007	1545	21	1.14
2008	1442	20	1.38
2009	1483	13	0.87
2010	1286	12	0.93
2011	1129	18	1.59
